# A longitudinal study on the correlation between postoperative complications and frailty in older patients with joint disorders

**DOI:** 10.1007/s40520-025-03126-0

**Published:** 2025-07-09

**Authors:** Yan Li, Juan Du, Liu He, Ying Chen, Lili Liu, Han Yan

**Affiliations:** 1 Department of Nursing, The Second Affiliate Hospital of Chengdu Medical College, Nuclear Industry 416 Hospital, Chengdu, 610051 China; 2 Faculty of Medicine, Bioscience and Nursing, MAHSA University, Bandar Saujana Putra, Jenjarom, 42610 Selangor Malaysia; 3 Department of Cardiothoracic Surgery, The Second Affiliate Hospital of Chengdu Medical College, Nuclear Industry 416 Hospital, Chengdu, 610051 China; 4 Department of Orthopaedics, The Second Affiliated Hospital of Chengdu Medical College, Nuclear Industry 416 Hospital Chengdu, Chengdu, 610051 China; 5 Department of Spine, The Second Affiliated Hospital of Chengdu Medical College, Nuclear Industry 416 Hospitall, Chengdu, 610051 China

**Keywords:** Frailty, The aged, Joint disorders, Postoperative complications, Longitudinal Study

## Abstract

**Aim:**

This study aimed to explore the effect of frailty on postoperative complications in older patients with joint disorders and to examine the trajectory of frailty changes within three months post-surgery.

**Methods:**

Older patients who were admitted for joint surgery from July to December 2023 were selected as study subjects. Data collected included general patient information, frailty scores, Age-adjusted Charlson Comorbidity Index (ACCI), Barthel Index and postoperative complications within 30 days after surgery. Frailty assessments were repeated at 1, 2, and 3 months post-surgery.

**Results:**

The incidence of postoperative complications was 32.5% and Frailty and ACCI were independent risk factors. The area under the curve (AUC) for predicting postoperative complications using frailty, ACCI, and their combination were 0.764, 0.747, and 0.814 respectively, with the combination showing superior predictive ability compared to ACCI alone (*P* < 0.05). Significant time and group effects were observed in frailty levels at the 1-, 2-, and 3-month postoperative time points between the complication and non-complication groups (*P* < 0.001), while the time-group interaction effect was not significant (*P* = 0.643).

**Conclusion:**

Frailty serves as a valuable auxiliary predictor of postoperative complications in older patients with joint disorders.During the first 1–3 months after surgery, both the complication and non-complication groups exhibited similar declining trends in frailty levels but the former consistently showed higher frailty at each time point. Emphasis on frailty management is essential throughout the perioperative period and key stages of postoperative rehabilitation, with a dual focus on symptom management and frailty intervention in patients with complications to promote recovery.

## Introduction

With aging, joint elasticity in older adults gradually decreases, heightening the risk of degenerative osteoarthritis, fractures, and other conditions that significantly impact quality of life [1].Surgery is a common treatment for joint disorders; however, older patients face increased risks of complications due to reduced physical function and tolerance to surgery, making comprehensive preoperative assessment essential.

Frailty, a pathological state characterized by reduced physical resilience and diminished capacity to cope with stress due to the accumulation of multiple deficits, is a global clinical and public health challenge [2].Studies have identified frailty as an independent risk factor for 30-day and 180-day postoperative mortality across seven emergency general surgery (EGS) procedures including laparotomy, surgical treatment of ulcer of stomach or duodenum, lysis of adhesions, excision of small intestine, appendectomy, colectomy, and cholecystectomy [3]. In patients over 80 years old, frailty can even serve as a basis for opting against surgery [4].

However, surgery itself poses a stressor that may trigger or exacerbate frailty [5]. Frailty is a continuously changing state, adding complexity to nursing care. Current research rarely focuses on the dynamic changes in frailty, particularly during the postoperative recovery period [6, 7].Studies have indicated that these longitudinal changes are associated with short-term mortality in older adults [8] and significantly affect joint function, where increased frailty may lead to reduced postoperative Oxford Hip/Knee Score (OHS/OKS) in patients undergoing hip or knee arthroplasty [9].Therefore, this study aims to explore the effect of frailty on postoperative complications in older patients with joint disorders through a longitudinal approach, while also tracking dynamic changes in frailty levels during the recovery process. The findings are expected to provide insights for clinical nursing decisions and community geriatric care.

## Methods

### Study design and sampling

This prospective longitudinal study enrolled older patients undergoing elective surgery for degenerative or traumatic joint disorders at Nuclear Industry 416 Hospital between July and December 2023, using convenience sampling. The included conditions were primarily osteoarthritis, post-traumatic arthritis, chronic joint instability, lumbar spinal stenosis, degenerative spondylolisthesis, and other related disorders. Surgeries included joint replacement, arthroplasty, internal fixation, excision, and other procedures such as ligament reconstruction, spinal decompression, and spinal fusion.The study was approved by the Ethics Committee of The Second Affiliate Hospital of Chengdu Medical College, Nuclear Industry 416 Hospital (YJ-2023-035).

### Inclusion and exclusion criteria of participants

The inclusion criteria were follows:1) ≥ 60 years old; 2) undergoing their first orthopedic surgery; 3)volunteered to participate in this study. The exclusion criteria were:1) presence of malignant tumors with extensive metastasis; 2) comorbid autoimmune diseases or mental disorders; 3) history of other surgical treatments within the past three months.

The sample size was calculated as no less than 109 totally (considering α of 0.05, allowable error of 10%), including a loss to follow-up rate of 20% based on the sample size calculation formula $n = {\left( {\frac{{{Z_{1 - \alpha /2}}}}{\delta }} \right)^2}p\left( {1 - p} \right)$ and referencing the complication rate reported in the literature [9]. Ultimately, 124 patients were included in the study.

### Outcome measures

#### Observational index

General information collected included gender, age, marital status, living situation, smoking history, drinking history, body mass index(BMI), and other relevant details.

Frailty was measured using the Groningen Frailty Indicator (GFI), which was developed by Dutch scholar Steverink and colleagues [10] and is widely used to assess frailty in community-dwelling and institutionalized older adults. The GFI consists of 15 items covering four domains: physical, cognitive, psychological, and social. A total score of ≥ 4 is considered indicative of frailty. The tool has demonstrated good internal consistency (Cronbach’s α = 0.712) and excellent test-retest reliability (*r* = 0.939). Unlike other tools such as the Fried Phenotype or Frailty Index, the GFI does not require physical performance testing or complex clinical evaluations, making it practical and feasible for use in older adults undergoing elective orthopedic surgery. It is also well suited for repeated administration in longitudinal studies.

Comorbidity Index was Assessed with the Age-adjusted Charlson Comorbidity Index (ACCI) [11], with the final score calculated based on weighted scores for 19 different diseases and various age groups.

Activities of Daily Living (ADL) was evaluated with the Modified Barthel Index (BI), which includes 10 items such as eating, personal hygiene, and toileting. The total score is 100 points, with higher scores indicating greater ADL independence.The scale has a Cronbach’s α coefficient of 0.810 [12].

Postoperative complications in this study were defined as any adverse events occurring within 30 days after surgery, including inpatient complications, outpatient diagnoses, and hospital readmissions [13]. The types of complications recorded included deep vein thrombosis, poor wound healing, wound infection, urinary tract infection, pulmonary infection, hypoproteinemia, and anemia. Anemia was defined as hemoglobin < 13 g/dL in males and < 12 g/dL in females, according to WHO criteria, and hypoproteinemia was defined as serum albumin < 35 g/L. For both conditions, clinical significance was further evaluated based on the need for treatment, such as blood transfusion, iron supplementation, or albumin infusion.Both were documented regardless of whether treatment was administered. Cases that required clinical intervention—such as albumin infusion or blood transfusion—were graded using the Clavien–Dindo system, while those without intervention were recorded separately for observational analysis.Participants are then categorized into a complications group and a non-complications group.

### Data collection

A research team was established, and members underwent standardized training prior to conducting patient surveys and follow-ups. Data were collected at five time points: at admission (T0), one day before discharge (T1), one month after discharge (T2), and at two months (T3) and three months (T4) post-discharge. At T0 and T1, data collection was conducted through face-to-face interviews combined with medical record reviews. T0 included baseline demographics, comorbidities, preoperative frailty (GFI), and admission laboratory indicators. T1 focused on inpatient complications prior to discharge. Post-discharge follow-ups at T2, T3, and T4 were conducted during the last week of each respective month and included assessments of postoperative frailty status and any complications that occurred after discharge.

### Statistical analysis

Statistical analysis was performed using SPSS 27.0 software(IBM, Armonk, NY, USA).According to the Central Limit Theorem, with a sample size of 120, the distribution of the means can be approximated as normal.Therefore, in this study, continuous variables were presented as mean ± standard deviation ($\overline x \pm S$) and the independent *t*-tests were used for group comparisons.Categorical data were described by frequency and percentage(n,%) and chi-square test was employed for group comparisons.The predictive efficacy of frailty for postoperative complications was analyzed using the receiver operating characteristic (ROC) curve. The differences in frailty scores at different time points were compared using repeated measures analysis of variance(ANOVA). A *p-value* of < 0.05 was considered statistically significant.

## Results

### Characteristics of the participants

During the study, two patients died, and two patients declined follow-up. Ultimately, 120 patients completed follow-up, including 38 males and 82 females, with an average age of 73.47 ± 8.48 years. Preoperative frailty scores of the participants averaged 4.84 ± 2.79, with 78 patients (65%) classified as frail. Within 30 days postoperatively, 15 patients transitioned from non-frail to frail status. A total of 39 patients (32.5%) experienced postoperative complications, including deep vein thrombosis(DVT) (5 cases), poor wound healing (7 cases), wound infection (8 cases), urinary tract infection (4 cases), pulmonary infection (6 cases), hypoproteinemia (11 cases), and anemia (12 cases).

### Analysis of factors influencing postoperative complications

Univariate analysis results showed that age, ACCI, frailty, and ADL were associated with the occurrence of complications. Multicollinearity testing showed that all four factors had tolerances > 0.1 and variance inflation factors(VIFs) < 10, suggesting no significant multicollinearity. A binary logistic regression analysis revealed that ACCI and preoperative frailty were independent risk factors for complications (Tables 1 and 2).

**Table 1:** Analysis of factors influencing postoperative complications

Indicators	Complications Group, *n* = 39	Non-Complications Group, *n* = 81	t/χ^2^	*P*
Sex, n(%)			1.233	0.267
Male	15(38.5)	23(28.4)		
Female	24(61.5)	58(71.6)		
Age(years)	77.49 ± 9.23	71.54 ± 7.40	3.514	<0.001
Marital Status, n(%)			1.239	0.266
With a spouse	33(84.6)	74(91.4)		
Without a spouse	6(15.4)	7(8.6)		
Educational level, n(%)			4.346	0.226
Primary school and below	18(46.2)	34(42.0)		
Middle school	12(30.8)	32(39.5)		
High school	4(10.3)	12(14.8)		
Junior college or higher	5(12.8)	3(3.7)		
Living situations, n(%)			2.417	0.299
With family	35(89.8)	74(91.4)		
With people other than family	3(7.7)	2(2.5)		
Living alone	1(2.6)	5(6.2)		
Joint Location, n(%)			4.170	0.383
Hip Joint	10(25.6)	14(17.3)		
Knee Joint	5(12.8)	12(14.8)		
Spinal Joint	19(48.7)	36(44.4)		
Shoulder Joint	5(12.8)	13(16.0)		
Other Small Joints	0	6(7.4)		
Body Mass Index (BMI)	23.28 ± 3.88	23.79 ± 2.68	-0.828	0.409
ACCI	6.31 ± 2.18	4.49 ± 1.50	4.692	<0.001
Frailty score	6.49 ± 2.74	3.98 ± 2.39	5.473	<0.001
ADL	57.95 ± 21.36	70.68 ± 21.30	-3.064	0.003
Smoking Status, n(%)			0.342	0.559
Yes	3(7.7)	9(11.1)		
No	36(92.3)	72(88.9)		
Alcohol Consumption Status, n(%)			0.020	0.888
Yes	4(10.3)	9(11.1)		
No	35(89.7)	72(88.9)		

Note: Frailty values refer to frailty measured at T0 (admission)

**Table 2:** Multifactorial analysis of postoperative complications

Indicators	B	SE	Waldχ2	*P*	OR	95CI
Constant	-3.577	2.463	2.109	0.146	0.028	
Age	-0.009	0.035	0.063	0.802	0.991	0.926∼1.061
Frailty	0.285	0.128	4.975	0.026	1.330	1.035∼1.708
ACCI	0.474	0.159	0.424	0.003	1.607	1.176∼2.195
ADL	-0.009	0.013	0.429	0.513	0.991	0.966∼1.018

Note: Frailty values refer to frailty measured at T0 (admission)

### The predictive value of frailty for postoperative complications

**Fig. 1:**
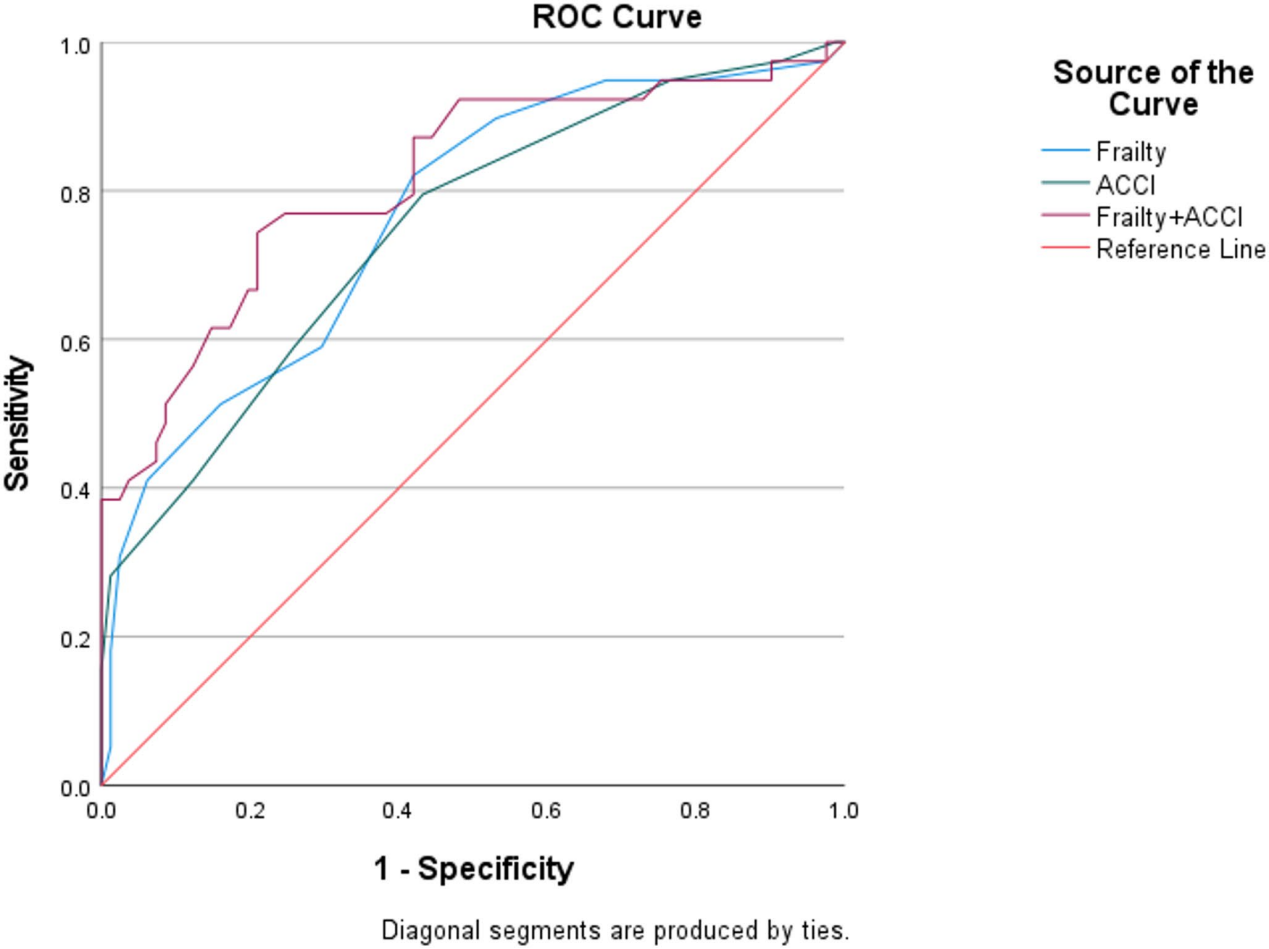
ROC curves of various indicators for predicting postoperative complications

The ROC curve was used to evaluate the predictive value of frailty, ACCI and their combined use for the occurrence of postoperative complications. The AUC values were 0.764, 0.747, and 0.814, respectively. The z-test showed that frailty has a predictive effectiveness for postoperative complications in older patients with joint disorders comparable to that of ACCI (*P* > 0.05). The combined use of frailty and ACCI did not show a significant difference in predictive effectiveness compared to frailty alone (*P* > 0.05) but was superior to using ACCI alone (*P* < 0.05)( Fig. 1; Tables 3 and 4).

**Table 3:** ROC curve analysis of different indicators for predicting postoperative complications

Indicators	Cut-off Value	AUC	Sensitivity	Specificity	Youden index	95%CI	*P*
ACCI	4.5	0.747	79.49%	56.79%	0.36	0.654–0.841	<0.001
Frailty	4.5	0.764	82.05%	58.03%	0.40	0.672–0.856	<0.001
Frailty + ACCI	-	0.814	74.36%	79.01%	0.53	0.727–0.902	<0.001

Note: Frailty values refer to frailty measured at T0 (admission)

**Table 4:** Comparison of the efficacy of different indicators in predicting postoperative complications

Indicators	z	*P*	SE	95%CI
Frailty—ACCI	0.328	0.743	0.305	-0.083 ~ 0.117
Frailty—Frailty + ACCI	-1.845	0.065	0.297	-0.104 ~ 0.003
ACCI—Frailty + ACCI	-2.176	0.030	0.299	-0.128~-0.007

Note: Frailty values refer to frailty measured at T0 (admission)

### Longitudinal tracking of frailty levels in older patients with joint disorders after surgery

**Fig. 2:**
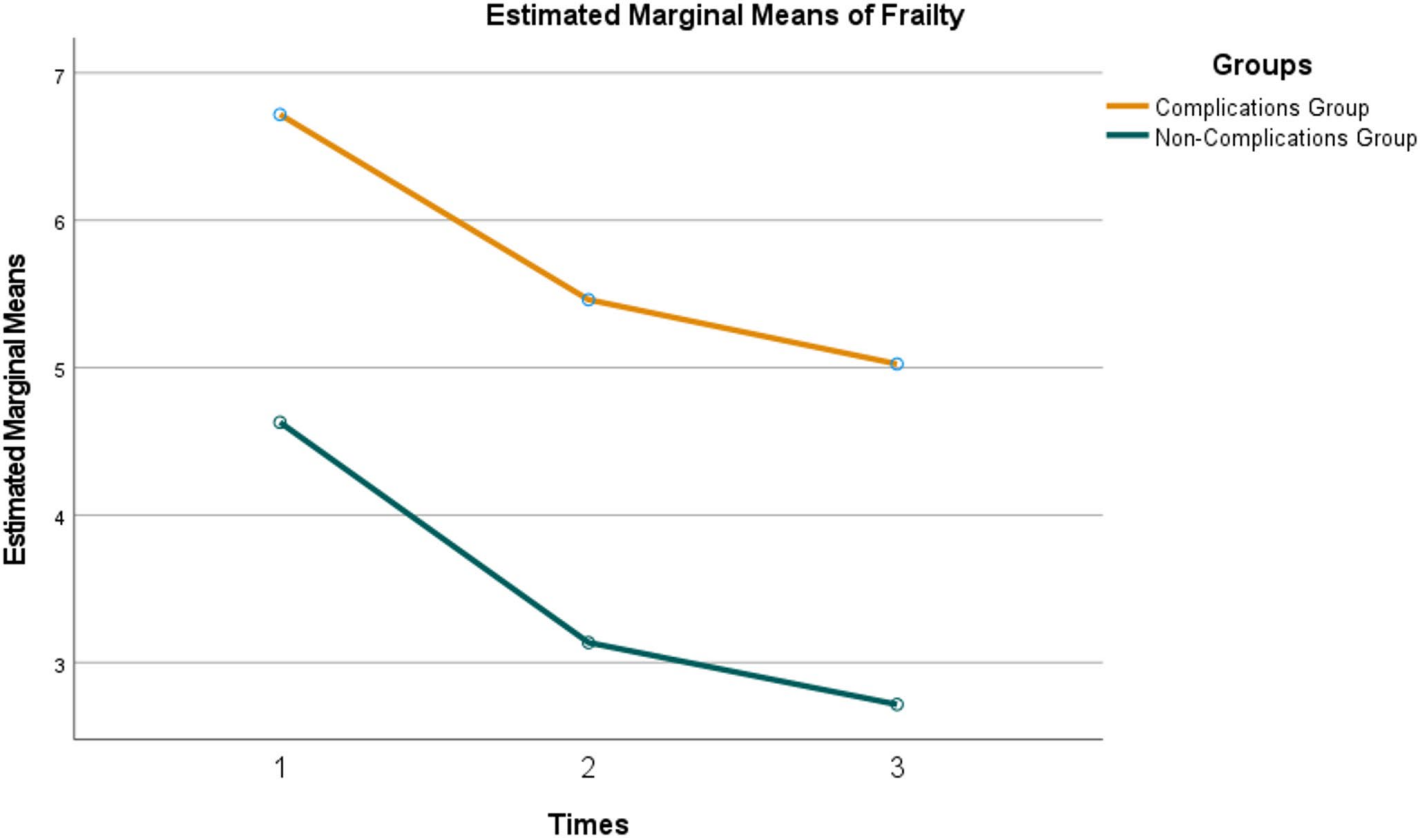
Changes in postoperative frailty levels in both groups of patients

A repeated measures ANOVA was conducted on patients’ frailty levels at T2, T3, and T4. The time effect was statistically significant, with frailty levels in both groups continuously decreasing at T2, T3, and T4 ( *P* < 0.001). The group effect was also statistically significant, with frailty levels in the complications group being higher at each time point compared to the non-complications group (*P* < 0.001). The interaction effect between time and group was not significant, indicating a similar trend in frailty level changes in both groups (*F* = 0.336, *P* = 0.643)(Table 5; Fig. 2).

**Table 5:** Comparison of frailty levels between two patient groups at different time points

Groups	T2	T3	T4	F	*P*
Complications group, *n* = 39	6.90 ± 3.04	5.64 ± 3.32^*^	5.21 ± 3.41^*#^	15.664	<*0.001*
Non-Complications group, *n* = 81	4.63 ± 3.25	3.14 ± 2.80^*^	2.72 ± 2.68^*#^	35.295	<*0.001*
*t*	3.651	4.068	3.999		
*P*	<*0.001*	<*0.001*	<*0.001*		
Groups	*F* = 18.596,*P*<*0.001*				
Time	*F* = 67.808,*P*<*0.001*				
Groups*Time	*F* = 0.336,*P =* 0.643				

Note: Compared with T2, *P < 0.001; compared with T3, ^#^*P* < 0.001

## Discussion

### High incidence of short-term postoperative complications in older patients with joint disorders

In this study, the incidence of postoperative complications in older patients with joint disorders was 32.5%, consistent with findings from similar studies [14]. The complications observed included infections, DVT, poor wound healing, hypoproteinemia, and anemia. These align with previous research [15]and are likely due to factors such as significant blood loss during surgery, the limited compensatory capacity in older patients, and the impact of pain from the disease and surgery on dietary intake. Complications like Venous thromboembolism (VTE) and infections can prolong hospital stays, increase treatment costs, and pose serious health risks, potentially even life-threatening ones [16].These risks have garnered widespread attention, with the American College of Chest Physicians’ guidelines for VTE categorizing major orthopedic surgery patients as high-risk and recommending routine pharmacological and physical interventions [17].

Notably, despite the routine administration of prophylactic anticoagulants (low molecular weight heparin, LMWH), five cases of deep vein thrombosis (DVT) were identified in our study. Among them, only one patient exhibited clinical symptoms such as lower-limb pain and swelling, while the remaining four cases were asymptomatic and were detected during scheduled lower-limb Doppler ultrasound examinations performed as part of our routine follow-up protocol. Although these thrombi may not have had an immediate clinical impact, they nonetheless represent a potential risk to patient safety. This observation suggests that for patients undergoing joint surgery—particularly those identified as frail in the preoperative period—routine ultrasound screening prior to discharge or during the early postoperative phase may be beneficial. Such a proactive approach could facilitate early detection and timely intervention, rather than relying solely on symptom-triggered evaluations.

### Frailty as a significant predictor of short-term postoperative complications in older patients with joint disorders

The study findings indicated that frailty and ACCI were independent risk factors for postoperative complications in older patients with joint disorders. Among these, an ACCI score of ≥ 5 was a strong predictor of postoperative complications, consistent with previous findings [15]. Recently, researchers have introduced frailty into this field, observing that burn patients with frailty have higher risks of complications, including myocardial infarction, sepsis, and urinary tract infections [18]. Surgery often disrupts endocrine, metabolic, immune functions, and frail patients are more susceptible to complications due to reduced reserves across multiple physiological systems, which decreases their ability to withstand stress and maintain stability [19]. The occurrence of wound complications, such as infections and poor healing, is closely related to lower physiological reserves and poor nutritional status [20].

In this study, ROC curve analysis demonstrated that frailty was comparable to ACCI in predicting postoperative complications in older patients with joint disorders (*P* > 0.05). Combining frailty and ACCI improved predictive accuracy compared to using ACCI alone (*P* < 0.05), suggesting that adding a frailty assessment to existing evaluations could better predict the likelihood of postoperative complications.Patients with joint diseases are often older and suffer from chronic pain, mobility impairment, even disability, and are unable to take care of themselves. This somatic disorder and the negative emotions it generates may also increase the risk of frailty [21, 22].Therefore, it is advisable to include frailty in preoperative assessment or to add frailty assessment to the existing assessment to better identify high-risk groups for complications.

This study identified 4.5 as the optimal GFI cutoff for predicting postoperative complications, suggesting that patients with a GFI score ≥ 5 are at particularly high risk and should receive special attention in perioperative management. While the original GFI scale defines frailty as a score of 4 or above, our findings refine this threshold in the context of complication prediction, without contradicting the established frailty definition. It emphasizes that among frail individuals, those with higher GFI scores may require more intensive preventive strategies. This may also be influenced by the specific characteristics of our study population. Therefore, prehabilitation measures—including nutritional supplementation and psychological support prior to surgery—are recommended to mitigate risk and enhance postoperative recovery outcomes [23–25].

### The complication group consistently exhibited higher levels of frailty during the postoperative recovery phase

Frailty has been shown to increase the risk of adverse outcomes, as demonstrated in previous studies [26, 27]. Surgical trauma itself acts as a stressor, and factors such as excessive postoperative sedation, premature nutritional therapy, and postoperative immobilization may lead to acquired frailty [22]. Research indicated that frailty was a predictor of long-term mortality following emergency surgery [5]. While over time, patients may gradually develop frailty under the influence of various negative factors. In elderly individuals, longitudinal changes in frailty are associated with short-term mortality rates. In this study, longitudinal observation revealed a gradual decline in frailty levels at three postoperative time points, yet frailty levels in the complication group remained consistently higher than those in the non-complication group.

Frailty not only increased the risk of adverse outcomes, such as falls and mortality in the elderly, but also negatively impacted postoperative joint function recovery [28, 29]. Therefore, for patients who develop postoperative complications, symptom management should be accompanied by frailty management. Healthcare providers, community health workers, and family caregivers should support patients in actively addressing these challenges on multiple fronts to promote postoperative recovery. Some studies have shown that activities like slow walking, resistance exercises, and enhanced enteral nutrition can improve physical status in older adults and slow the progression of frailty [30]. Healthcare providers, community health workers, and family caregivers can assist patients by promoting these measures. It is noteworthy that although no statistically significant difference was observed in GFI score changes between the two groups, the non-complications group showed a trend toward a greater decline in mean GFI scores compared to the complications group. Whether complications influence long-term frailty levels remains to be clarified through extended longitudinal research.

This study has several strengths. Its prospective longitudinal design enabled us to assess both the predictive role of preoperative frailty and the dynamic changes in frailty after surgery. Complications were comprehensively recorded, including asymptomatic events detected through active screening. The use of the Groningen Frailty Indicator allowed for a multidimensional assessment of patients’ vulnerability. However, some limitations should be acknowledged. The study was conducted in a single center with a relatively small sample size, which may affect the generalizability of the results. In addition, the follow-up period was limited to three months, and long-term functional and clinical outcomes were not assessed. Further multicenter studies with longer follow-up are warranted to confirm and expand upon these findings.

## Conclusions

This study conducted a longitudinal investigation into the association between frailty and postoperative complications in older adults undergoing surgery for joint disorders. The results confirmed that preoperative frailty is an independent risk factor for postoperative complications and has comparable predictive accuracy to the ACCI, with their combination offering improved predictive value. These findings highlight the clinical value of integrating frailty screening into routine preoperative assessments to identify high-risk patients and guide targeted interventions.Notably, both the complication and non-complication groups exhibited a significant decline in frailty scores over the first three months postoperatively, although the complication group consistently maintained higher frailty levels. While no significant difference was observed in the downward trend of frailty between the two groups during the early postoperative period, whether the occurrence of complications influences frailty recovery in the longer term remains to be determined in future studies.

## Data Availability

Data from this study are not publicly available because of patient privacy, but readers are invited to contact the authors if necessary.
